# Blocking Mineralocorticoid Receptors prior to Retrieval Reduces Contextual Fear Memory in Mice

**DOI:** 10.1371/journal.pone.0026220

**Published:** 2011-10-12

**Authors:** Ming Zhou, Merel Kindt, Marian Joëls, Harm J. Krugers

**Affiliations:** 1 Center for Neuroscience, Swammerdam Institute for Life Sciences, University of Amsterdam, Amsterdam, The Netherlands; 2 Department of Clinical Psychology, University of Amsterdam, Amsterdam, The Netherlands; 3 Department of Neuroscience and Pharmacology, Rudolf Magnus Institute of Neuroscience, University Medical Center Utrecht, Utrecht, The Netherlands; University of Minnesota, United States of America

## Abstract

**Background:**

Corticosteroid hormones regulate appraisal and consolidation of information via mineralocorticoid receptors (MRs) and glucocorticoid receptors (GRs) respectively. How activation of these receptors modulates retrieval of fearful information and the subsequent expression of fear is largely unknown. We tested here whether blockade of MRs or GRs during retrieval also affects subsequent expression of fear memory.

**Methodology/Principal Findings:**

Mice were trained in contextual or tone cue fear conditioning paradigms, by pairing mild foot shocks with a particular context or tone respectively. Twenty-four hours after training, context-conditioned animals were re-exposed to the context for 3 or 30 minutes (day 2); tone-conditioned animals were placed in a different context and re-exposed to one or six tones. Twenty-four hours (day 3) and one month later, freezing behavior to the aversive context/tone was scored again. MR or GR blockade was achieved by giving spironolactone or RU486 subcutaneously one hour before retrieval on day 2. Spironolactone administered prior to brief context re-exposure reduced freezing behavior during retrieval and 24 hours later, but not one month later. Administration of spironolactone without retrieval of the context or immediately after retrieval on day 2 did not reduce freezing on day 3. Re-exposure to the context for 30 minutes on day 2 significantly reduced freezing on day 3 and one month later, but freezing was not further reduced by spironolactone. Administration of spironolactone prior to tone-cue re-exposure on day 2 did not affect freezing behavior. Treatment with RU486 prior to re-exposure did not affect context or tone-cue fear memories at any time point.

**Conclusions/Significance:**

We conclude that MR blockade prior to retrieval strongly reduces the expression of contextual fear, implying that MRs, rather than GRs, play an important role in retrieval of emotional information and subsequent fear expression.

## Introduction

Memories for emotionally arousing and stressful events are generally well retained [1]. If sufficiently stressful, these events activate the Hypothalamus-Pituitary-Adrenal (HPA)-axis which increases the release of corticosteroid hormones from the adrenal glands [2]. Corticosteroid hormones readily cross the blood brain barrier and bind to high affinity mineralocorticoid receptors (MRs) and lower affinity glucocorticoid receptors (GRs) [3]. Upon binding to their receptors, corticosteroid hormones regulate and promote distinct phases of learning and memory processes. Several studies have shown that post-training activation of GRs promotes consolidation of fearful information [4], [5], [6], [7], [8]. Activation of MRs is critical for the appraisal of stressful information and response selection [7], [9], [10]. In addition, genetic deletion of forebrain MRs hampers spatial learning [11] and pharmacological blockade of MRs impairs contextual fear conditioning [8], [11].

Surprisingly little is known about how corticosteroid hormones and their receptors regulate the retrieval of fearful information. While exposure to stressful experiences and elevated corticosteroid hormones has been reported to suppress the retrieval of spatial information [12], [13], it remains to be investigated whether activation of MRs and GRs by endogenously released corticosteroid hormones is involved in this process. Regulation of retrieval and subsequent (re)consolidation by MRs and/or GRs might potentially take place for at least two reasons. First, retrieval of fearful information is a stressful event in itself and accompanied by elevated corticosteroid hormone levels [14]. Second, retrieval and re-activation of fearful events renders these memories labile and protein synthesis is required after reactivation to re-consolidate the memory trace [15]. Reconsolidation has been demonstrated in various tasks and species [15], [16], [17], including humans [18], [19]. The notion that stored memories can be turned into a labile state has opened new avenues to reduce excessive fears more permanently than the traditional extinction procedure. For example, treatment with β-adrenergic receptor antagonists during re-exposure has been reported to affect the subsequent expression of fear for a considerable period of time [19], [20], [21]. Given that corticosteroid hormones, via activation of MRs and GRs, are potent regulators of fearful memories, we explored here whether blocking MRs and GRs during retrieval of a fearful context or tone regulates the subsequent expression of fearful memories. We report that MRs but not GRs regulate retrieval of fearful information.

## Results

### MR blockade prior to brief context re-exposure reduces fear expression

**Figure 1:**
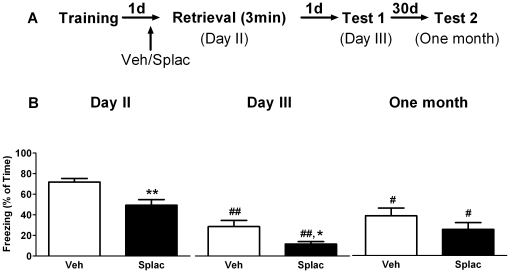
MR blockade prior to brief context re-exposure reduces subsequent fear expression. A) Behavioral procedure for the experiment. B) Freezing behavior in both vehicle and spironolactone treated mice decreased both on day 3 and one month later when compared to day 2. Treating mice with spironolactone one hour before brief (3 minutes) retrieval (day 2) reduced freezing behavior compared to vehicle treated mice, both during the retrieval session and one day later (day 3). **^#^** and **^##^** reflect P<0.05 and P<0.01 respectively when compared to day 2; *and**reflect P<0.05 and P<0.01 respectively when compared to vehicle treated mice at the same time point (n = 9 mice per group).

During training animals displayed a progressive increase in freezing behavior (repeated measures ANOVA, F_3, 99_ = 29.91, P<0.01). Importantly, freezing behavior during training was comparable for the groups that were later treated with vehicle or the MR-antagonist spironolactone (F_1, 33_ = 0.27, P>0.05). Twenty three hours later, animals received either spironolactone or vehicle injection. One hour after drug administration, animals were re-exposed to the same context as used during training and freezing behavior was measured at that time (day 2) as well as 24 hours (day 3) and one month later. In the first series of experiments we tested the effect of spironolactone given prior to brief (3 minutes) context re-exposure (Figure 1). Repeated measures ANOVA showed a main effect of time in both vehicle (F_2, 16_ = 17.87, P<0.01) and spironolactone treated animals (F_2, 16_ = 22.88, P<0.01). Multiple mean comparisons with *post hoc* Bonfferoni test revealed that both vehicle and spironolactone treated mice showed significantly less freezing behavior on day 3 (P<0.01) and one month later (P<0.05) respectively, when compared to day 2 (Figure 1B). Repeated measures ANOVA also revealed a main effect of treatment (F_1, 16_ = 9.92, P<0.01). *Post hoc* test showed that treatment with spironolactone reduced freezing behavior during retrieval (Day 2, P<0.01) as well as 24 hours after the retrieval trial (Day 3, P<0.05) when compared to vehicle treatment. No significant treatment effect was found one month after retrieval (P>0.05). These results indicate that spironolactone, when applied before a brief retrieval of contextual information reduces the expression of fear for at least one day.

**Figure 2:**
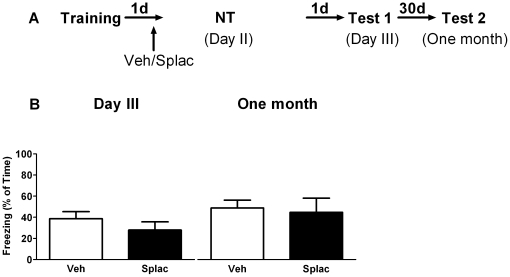
MR blockade without context re-exposure has no effect on subsequent fear expression. A) Behavioral procedure for the experiment. B) Spironolactone was injected 23 hours after training in the absence of re-exposure to the fearful context on day 2. No effect of spironolactone on contextual memory retention was found twenty four hours (day 3) or one month later (n = 6 mice per group).

**Figure 3:**
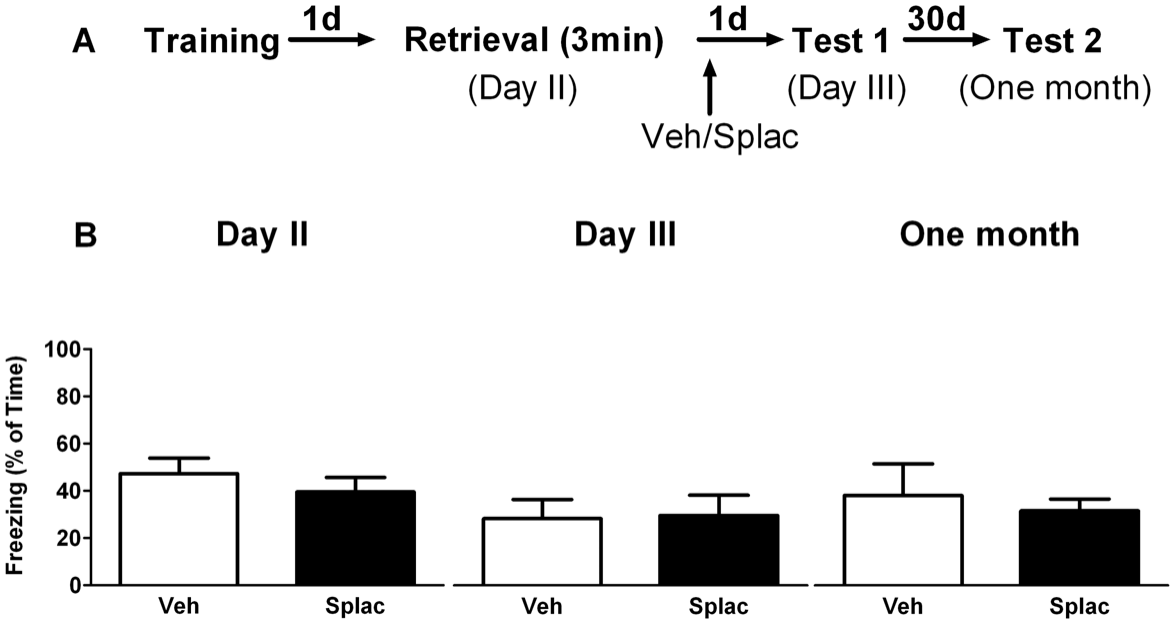
MR blockade immediately after brief context re-exposure has no effect on subsequent fear expression. A) Behavioral procedure for the experiment. B) Freezing behavior during brief retrieval was comparable between the two groups. Treating animals with spironolactone immediately after brief re-exposure did not affect contextual memory retention on day 3 or one month after retrieval (n = 6 mice per group).

In a second series of experiments spironolactone was applied 23 hours after training, but in the absence of re-exposure to the context (Figure 2A, NT). Under these conditions, no main effect of spironolactone treatment (Figure 2B, F_1, 10_ = 0.44, P>0.05) was observed. This indicates that spironolactone is only effective in reducing the expression of fear when the drug is present during retrieval of information. This was confirmed by another set of experiments where spironolactone was administered immediately after retrieval (Figure 3). In the vehicle-treated animals, no statistical difference in freezing scores was found over time (day 2, day 3 and one month later), by repeated measures ANOVA (within-group comparison). Nevertheless, a paired t-test revealed that animals froze significantly less on day 3 when compared to day 2 (P = 0.001), which is in agreement with the data presented in Figure 1, and confirms that brief retrieval of contextual information results in less freezing. No between-group effects of spironolactone on freezing behavior were found at any time point. Overall, these experiments reveal that blocking mineralocorticoid receptors reduces the expression of contextual fear for at least one day, but only when these receptors are blocked before/during retrieval.

**Figure 4:**
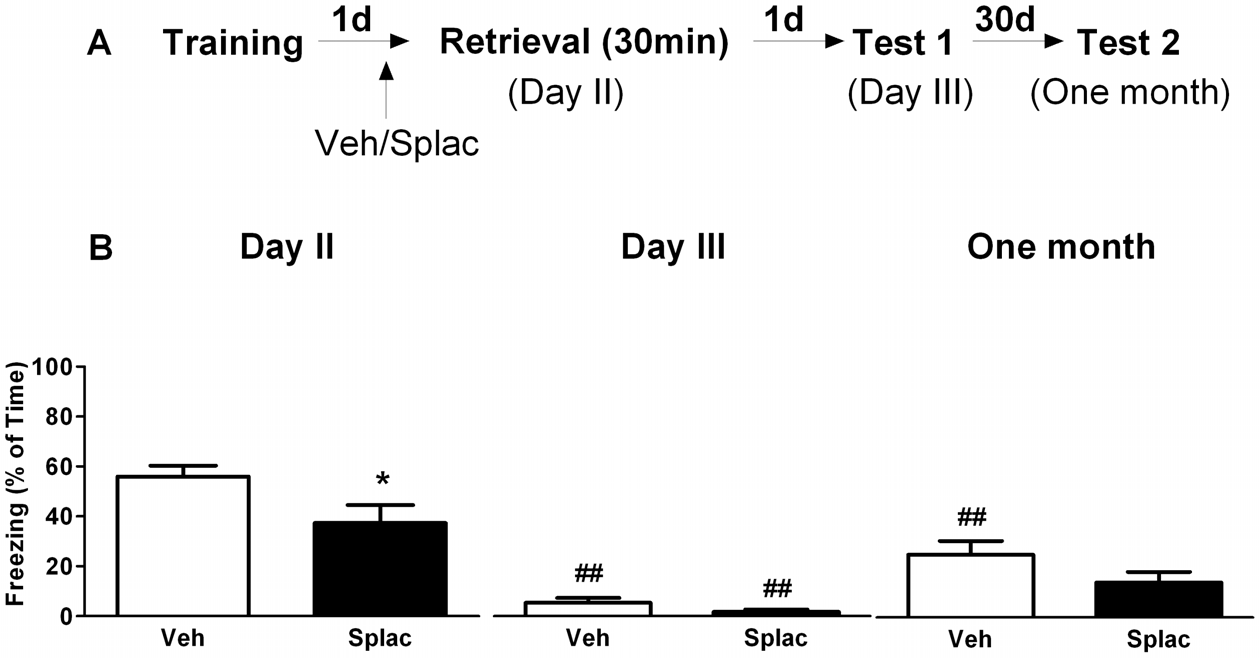
MR blockade prior to prolonged context re-exposure has no effect on subsequent fear expression. A) Behavioral procedure for the experiment. B) Freezing behavior decreased in both vehicle and spironolactone treated animals on day 3 and one month later only vehicle treated group showed difference in freezing scores, when compared to the first 3 minute re-exposure to the context on day 2. No between-group effect was found except for the first 3 minutes during prolonged retrieval on day 2. **^##^** reflects P<0.01 when compared to day 2. * reflects P<0.05 when compared to vehicle treated group (n = 8–9 mice per group).

Placing animals in the same context for 3 minutes is thought to initiate reactivation and subsequent reconsolidation, whereas placement in this context for 30 minutes supposedly promotes extinction learning [22]. To specifically explore the effect of spironolactone treatment on extinction of fear memories, a separate batch of animals was exposed to the context for 30 minutes on day 2, i.e. 24 hours after training (Figure 4A). We determined the freezing behavior during the first 3 minutes of the 30-minutes retrieval period and compared this with freezing scored on day 3 and one month later. Repeated measures ANOVA revealed a time effect in both vehicle (F_2, 16_ = 39.08, P<0.01) and spironolactone treated mice (F_2, 14_ = 11.85, P<0.01). *Post hoc* test showed (as expected) that compared to day 2, both vehicle and spironolactone treated animals displayed significantly less freezing on day 3 (P<0.01) though one month later only vehicle treated animals showed a significant reduction in freezing scores (P<0.01). A between group effect of spironolactone was also found (F_1, 15_ = 10.26, P<0.01). On day 2, spironolactone treated animals displayed less freezing during the first 3 minutes of the retrieval period when compared to vehicle treatment (Figure 4B, Day 2, P<0.05).

### MR blockade prior to tone cue re-exposure has no effect on fear expression

**Figure 5:**
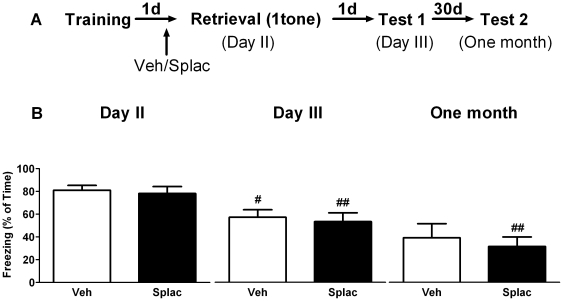
MR blockade prior to one tone re-exposure has no effect on subsequent fear expression. A) Behavioral procedure for the experiment. B) Freezing behavior decreased in only vehicle treated group on day 3 and in both groups one month later when compared to day 2. No significant spironolactone effect was found when compared to vehicle treated group. **^#^** and **^##^** reflect P<0.05 and P<0.01 respectively when compared to freezing on day 2 (n = 7 mice per group).

We next tested if spironolactone modifies the expression of fear when the drug is administered prior to retrieval of tone-cue memories. During training in context A, freezing behavior during and immediately after the tone was comparable between the groups that were later treated with vehicle or spironolactone (data not shown). One day after training, both vehicle and spironolactone treated mice displayed high levels of freezing in response to one tone in context B (Figure 5B). Data analysis indicated a main time effect in both vehicle (F_2, 12_ = 9.40, P<0.01) and spironolactone treated mice (F_2, 12_ = 35.59, P<0.01). When compared to day 2, both groups showed significant less freezing behavior on day 3 (vehicle: P<0.05; spironolactone: P<0.01) though one month later only spironolactone treated group showed less freezing (P<0.01), No between group-effect of spironolactone treatment was found (F_1, 12_ = 0.26, P>0.05).

**Figure 6:**
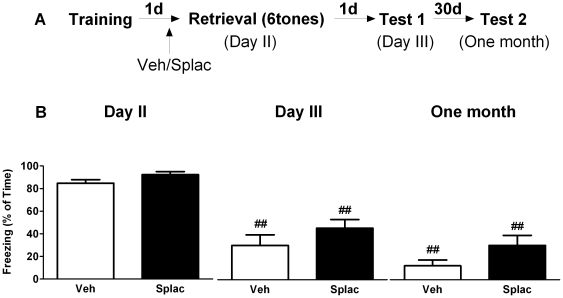
MR blockade prior to six-tone re-exposure has no effect on subsequent fear expression. A) Behavioral procedure for the experiment. B) Freezing behavior decreased in both vehicle and spironolactone treated groups on day 3 and one month later when compared to the first tone exposure on day 2. No differences were found between vehicle and spironolactone treatment. **^##^** reflects P<0.01 when compared to freezing on day 2 (n = 7 mice per group).

Repeated exposure to six tones on day 2 significantly decreased the freezing behavior over time during extinction training (repeated measures ANOVA, F_2,__24_ = 66.32, P<0.01). Freezing behavior was also scored one day (day 3) and one month later (Figure 6A). A significant within-subject effect was found over time in both vehicle (F_2, 12_ = 38.84, P<0.01) and spironolactone (F_2, 12_ = 27.98, P<0.01) treated mice; when compared to freezing behavior during the first tone on day 2, less freezing was scored both on day 3 (Figure 6B, P<0.01) and one month later (P<0.01) in both groups. No between-group effect of spironolactone was observed (F_1, 12_ = 4.71, P>0.05).

### GR blockade prior to context re-exposure has no effect on subsequent fear expression

**Figure 7:**
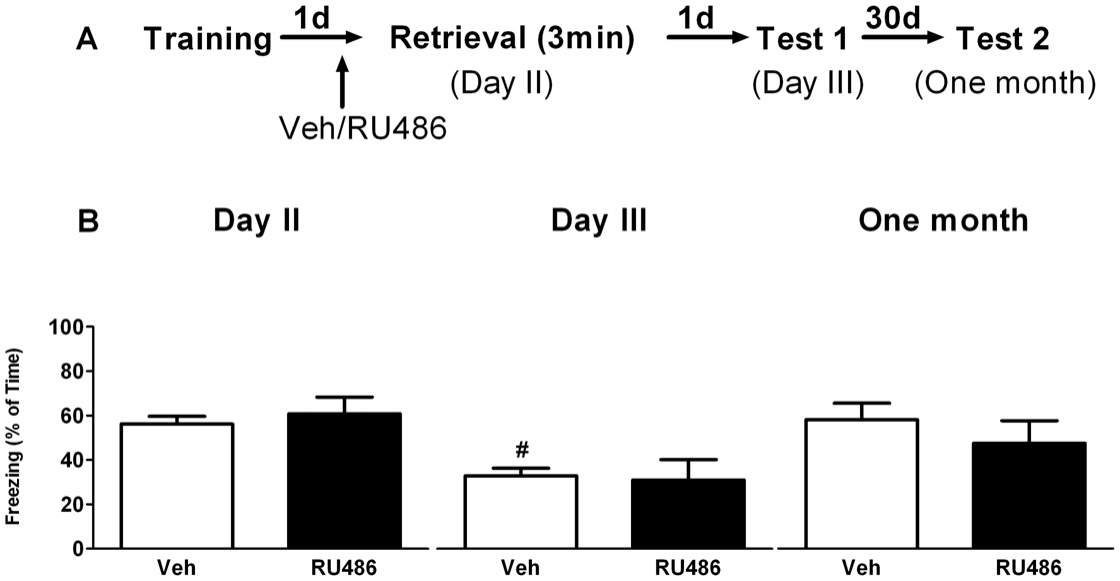
GR blockade prior to brief context re-exposure has no effect on subsequent fear expression. A) Behavioral procedure for the experiment. B) Freezing behavior decreased in only vehicle treated mice on day 3 compared to day 2. Treating mice with RU486 or vehicle (on day 2) resulted in comparable freezing behavior over time. **^#^** reflects P<0.05 when compared to freezing on day 2 (n = 7 mice per group).

During training animals displayed a progressive increase in freezing behavior (repeated measures ANOVA, F_3, 81_ = 31.61, P<0.01). No difference was found between the groups that were later treated with vehicle or the GR-antagonist RU486 (F_1, 26_ = 1.42, P>0.05). One hour after drug treatment on day 2, freezing behavior to the training context was measured for 3 minutes and then compared to that measured one day (day 3) or one month later. Repeated measures ANOVA showed a main time effect in both vehicle (Figure 7B, F_2, 12_ = 9.77, P<0.01) and RU486 (F_2, 12_ = 4.77, P<0.05) treated animals. *Post hoc* test revealed that, when compared to day 2, only vehicle treated animals displayed significantly less freezing on day 3 (P<0.01). No significant differences were found in spironolactone treated animals or one month later (P>0.05). Also no between-group effect of RU486 was found (F_1, 12_ = 0.11, P>0.05). These results suggest that activation of GRs is not critically involved in retrieval of contextual fearful information and subsequent expression of fear.

**Figure 8:**
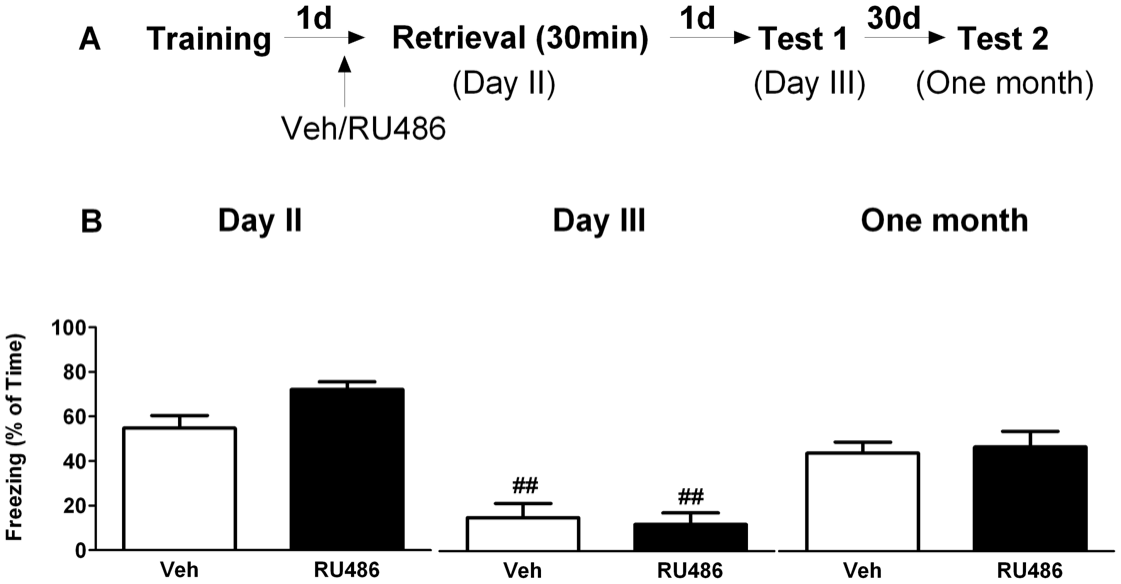
GR blockade prior to prolonged context re-exposure has no effect on subsequent fear expression. A) Behavioral procedure for the experiment. B) Freezing behavior in both vehicle and RU486 treated groups decreased on day 3 but not one month later when compared to the first 3 minute context re-exposure on day 2. No difference in freezing behavior was found between vehicle and RU486 treated groups. **^##^** reflects P<0.01 when compared to freezing on day 2 (n = 7 mice per group).

In a separate experiment animals were injected with RU486 or vehicle 23 hours after training and subsequently placed animals into the training context for 30 minutes; freezing behavior was scored on day 2, day 3 and one month later. Repeated measures ANOVA indicated a significant time effect in both vehicle (Figure 8B, F_2, 12_ = 26.29, P<0.01) and RU486 (F_2, 12_ = 40.83, P<0.01) treated mice. *Post hoc* test showed that compared to the first 3 min re-exposure on day 2, both groups showed a significant reduction in freezing behavior on day 3 (P<0.01) bu not (P>0.05). No between group effects of RU486 were found (F_1, 12_ = 0.002, P>0.05). These results suggest that GR activation is not critically involved in the effect of 30-minute retrieval process of contextual fear information and subsequent expression of fear behavior.

### GR blockade prior to tone-cue re-exposure has no effect on subsequent fear expression

**Figure 9:**
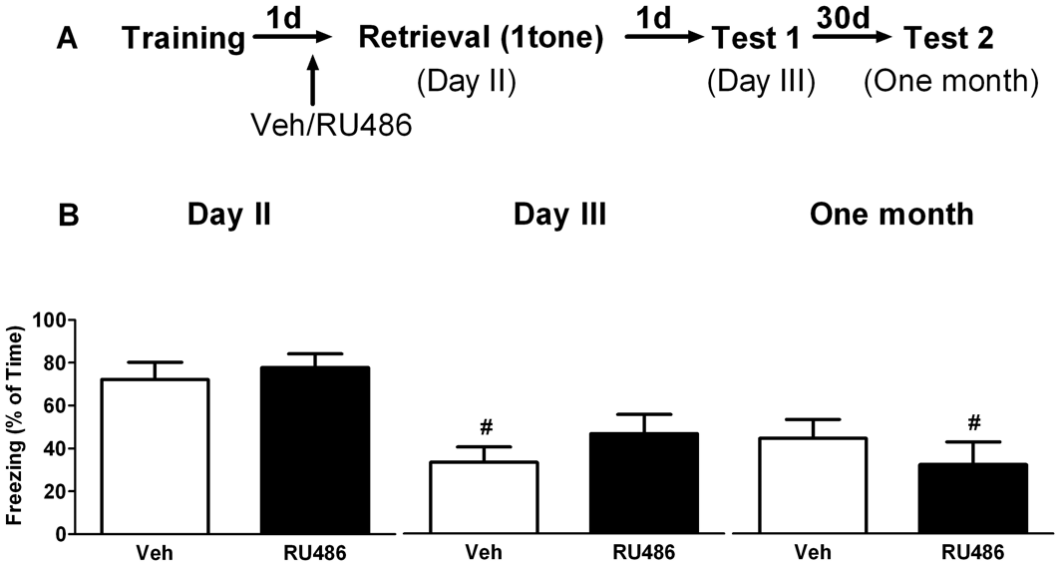
GR blockade prior to one tone re-exposure has no effect on subsequent fear expression. A) Behavioral procedure for the experiment. B) Freezing behavior in vehicle treated mice decreased on day 3 compared to day 2. Treatment with RU486 resulted in less freezing one month later compared to day 2. No significant RU486 effect was found. **^#^** reflects P<0.05 when compared to day 2 (n = 6 mice per group).

We next tested if RU486 affects the expression of tone-cue fear memory when administered prior to retrieval. During training, freezing behavior during and immediately after the tone was comparable between the mice that were later treated with either vehicle or RU486 (data not shown). Twenty-three hours later, mice were treated with either vehicle or RU486, and one hour later exposed to one tone. Freezing behavior was scored on day 2, day 3 and one month later respectively (Figure 9A). Repeated measures ANOVA revealed a significant time effect in both vehicle (Figure 9B, F_2, 10_ = 6.13, P<0.05) and RU486 (F_2, 10_ = 15.35, P<0.01) treated mice. *Post hoc* test revealed that vehicle treated group displayed a significant decrease in freezing behavior on day 3 compared to day 2 (P<0.05), while RU486 treated mice showed reduced freezing behavior one month later (P<0.05). No between group effect of RU486 treatment was found on tone-cue freezing (F_1, 10_ = 0.07, P>0.05).

**Figure 10:**
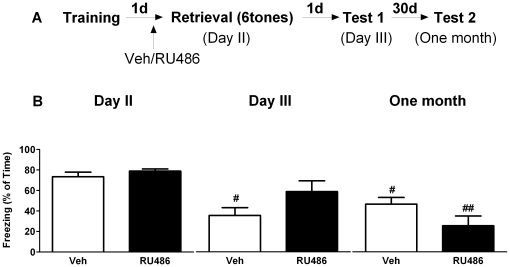
GR blockade prior to six-tone re-exposure has no effect on subsequent fear expression. A) Behavioral procedure for the experiment. B) Freezing behavior in vehicle treated mice decreased both on day 3 and one month later when compared to the first tone exposure on day 2. RU486 treated mice displayed reduced freezing one month later. No difference in freezing behavior was found between vehicle and RU486 treated animals. **^#^**and**^##^** reflect P<0.05 and P<0.01 respectively when compared to day 2 (n = 6 mice per group).

Repeated exposures to six tones on day 2 resulted in a significant decrease in freezing behavior (repeated measures ANOVA, F_2, 20_ = 23.93, P<0.01). Freezing behavior was further scored one day (day 3) and one month later (Figure 10A). A main time effect was found in both vehicle (F_2, 10_ = 12.87, P<0.01) and RU486 treated groups (F_2, 20_ = 17.29, P<0.01). Compared to day 2, vehicle group displayed less freezing behavior both on day 3 (Figure 10B, P<0.05) and one month later (P<0.05), while a reduction in freezing behavior was only seen in RU486 group one month later (P<0.01). No between group effect of RU486 treatment was found (F_1, 10_ = 0.11, P>0.05).

## Discussion

Corticosteroid hormones modulate distinct phases of learning and memory via activation of mineralocorticoid receptors (MRs) and glucocorticoid receptors (GRs). We tested here whether activation of MRs and GRs is also critical for retrieval and subsequent expression of fear. This is particularly interesting since retrieval of fearful events supposedly turns these memories into a labile state [17], [23], [24], which allows a window for pharmacological intervention. We found that blocking MRs reduces for at least one day the expression of fear after brief re-exposure to the fearful context, whereas extinction learning or tone-cue fear conditioning remained unaffected. Blocking GRs did not affect retrieval of information, extinction and subsequent expression of contextual and tone-cue fearful memories.

The protocol that we used to modulate the expression of fear–i.e. re-exposure on day 2 to the training context for 3 or 30 minutes-was previously described to examine reconsolidation and extinction of fearful memories respectively, which are two opposing processes that may be triggered by a similar memory retrieval procedure [22], [25]. These studies showed that brief retrieval of previously acquired fear induces reconsolidation, while prolonged re-exposure results in extinction. Our data show that activation of MRs during brief re-exposure but not prolonged re-exposure regulates the subsequent expression of fear. This could suggest that activation of MRs regulates reconsolidation of information. One might question, however, whether information was really reconsolidated or subject to weak extinction by brief re-exposure on day 2. We cannot exclude that MR blockade transiently facilitates a weak form of extinction learning as a significant spironolactone x re-exposure interaction effect was observed during and 24 hours after the manipulation. Inducing extinction learning with a stronger paradigm, i.e. by re-exposing the mice to the adverse context for 30 minutes, did not result in significant effects of spironolactone on freezing.

Our results demonstrate that MRs are critical during the retrieval of contextual emotional information for the subsequent expression of fear behavior, lasting at least one day but less than one month. Mineralocorticoid receptors (MRs) have been implicated in appraisal of novel and stressful events and response selection [7], [10]. If blocking MRs prior to retrieval affects response selection, we would predict only a temporary reduction of fear expression during the retrieval session, but a return of fear at the long-term. On the other hand, if blocking MRs upon retrieval affects reappraisal of the event, a more permanent fear reduction would be expected. Our current findings that spironolactone when administered prior to retrieval of information reduces freezing behavior during re-exposure as well as one day later (in the absence of the drug) but not one month later suggests that MRs are involved in response selection rather than re-appraisal of the fearful situation.

By blocking MRs, the relative contribution of GRs in behavioral effects might be increased as blocking MRs could have impaired the fast negative feedback of endogenous corticosterone on HPA-axis activity, leading to increased secretion of corticosterone [26]. This is relevant, since elevated corticosteroid levels have been reported to reduce retrieval of information and to promote the extinction of irrelevant information via GR activation [27], [28], [29]. The presumably stronger GR activation seems unlikely to explain the current findings, since 1) the effect of spironolactone observed here is not restricted to the retrieval process only, as it lasts at least till the next day; 2) spironolactone did not affect fear memory when given after re-exposure; and 3) RU 486 treatment at the time of retrieval did not affect any aspect of subsequent fear expression (see also below). Therefore, the effects of spironolactone are most likely the direct consequence of interfering with MR-mediated processes. This may involve direct modulation of relevant substrates for learning and memory such as plasticity at excitatory synapses [30]. More specifically, corticosteroid hormones increase hippocampal excitatory synaptic transmission via MRs [31], [32] and facilitate synaptic potentiation of these synapses via rapid non-genomic actions [33].

While administration of spironolactone prior to retrieval reduced the expression of contextual fear memory, tone-cue memory remained unaffected. This also strongly argues against a non-specific role of the drug in reducing fear expression. The differential effectiveness of spironolactone in context versus tone-cued memories could be explained by the MR distribution in areas playing a major role in these two types of memories, that is, the hippocampus and basolateral amygdala respectively. MRs are highly expressed in all hippocampal fields, whereas MR expression is much lower in the basolateral amygdala [2], [3]. Also, the efficacy of MRs to modulate synaptic transmission is different in amygdala [34] compared to the hippocampus [32].

Several studies have shown that post-training application of GR agonists promote (fearful) memory consolidation [5], [35], while GR antagonists suppress memory consolidation [7], [8], [10]. Our current results show that blocking GRs - at a dosage that is effective in hampering the consolidation of contextual fear – neither affected the retrieval of fearful information nor regulated the subsequent expression of fear. This may imply that GRs are critically involved in promoting the storage of fearful information, while they play a minor role in regulating already consolidated fearful information, even when these memories are labile. Nevertheless, a recent study described that post-retrieval application of RU486 (at a higher dosage than used in our current study) was effective in impairing memory reconsolidation in an inhibitory avoidance paradigm [36]. This may indicate that GRs, under particular but not all experimental conditions are able to regulate reconsolidation of memory.

In conclusion, our results demonstrate that blocking MRs, but not GRs, prior to retrieval of contextual fearful information reduces the expression of contextual fear later on, lasting for at least one day.

## Materials and Methods

### Animals

Male C57/BL6 mice (6–8 weeks old, Harlan, The Netherlands) were housed (2 mice per cage) for at least one week after arrival. All animals were kept on a light/dark cycle of 12 h (lights on at 8 a.m.; humidity 55%±15; room temperature kept at 22 °C ±2) and food and water were given without restriction. The experiments (training, memory retrieval and testing) were performed between 8:30–11:30 a.m. and approved by the local Animal Ethics Committee of the University of Amsterdam (DED212). All efforts were made to minimize suffering of the animals.

### Drugs and treatments

The mineralocorticoid receptor antagonist spironolactone (Splac, Sigma, 50 mg/kg), glucocorticoid receptor antagonist RU486 (Sigma, 10 mg/kg) or vehicle (Veh, propylene glycol) was injected subcutaneously one hour before the retrieval of contextual or tone-cued information (i.e. twenty three hours after training) or immediately after retrieval. The dosage and drug delivery route were chosen based on previous studies in which MR mediated effects were effectively blocked by the administration of MR antagonist [6], [37], [38] with little effect on spontaneous behavior [39], [40]. Similarly, GR-mediated effects could be prevented sufficiently with the current administration method, as documented previously [6].

### Contextual Fear Conditioning

Animals were trained in a fear conditioning chamber (Context A, W × L × H: 30 cm×24 cm×26 cm) that contained a grid floor with 37 stainless steel rods and was connected to a shock generator and sound generator (Med-Farm LION-ELD) developed in-house. During training, one animal at one time was placed into context A. After three minutes of free exploration, three foot shocks (2 seconds, 0.4 mA) were delivered with an interval of 90 seconds. Sixty seconds after the end of the last foot shock, the mouse was placed back into its home cage. Freezing behavior, defined as no body movements except those related to respiration, was determined every 2 seconds throughout the experiment [8], [41]. Twenty three hours later on day 2, mice were injected subcutaneously with either spironolactone, RU486 or vehicle and returned to their homecages. One hour later one animal at a time was placed in context A for either 3 or 30 minutes without receiving any foot shock, presumably initiating reconsolidation and extinction respectively based on previous studies [15], [22], [42]. Freezing behavior was scored throughout these periods. Twenty four hours (day 3, i.e. 48 hours after training) and one month later one animal at a time was placed in context A for 3 minutes without receiving foot shock and freezing behavior was scored.

In separate control experiments, animals received the injection with spironolactone or vehicle one day after training without or immediately after the retrieval of contextual information. Freezing behavior was monitored on day 2, day 3 and one month later.

### Tone-cued Fear Conditioning

All animals were handled for three days before the start of the experiments and placed for 20 minutes/day in context B which has the same size as context A, but different contextual background (odor, texture and color). During training, one mouse at a time was placed into context A. After three minutes of free exploration, the mouse was exposed to a tone (100 dB, 2.8 kHz) that lasted for 30 seconds and co-terminated with a mild foot shock (2 seconds, 0.4 mA) [8]. Thirty seconds later, the animal was placed back in its homecage. Twenty three hours later (day 2), mice were injected subcutaneously with spironolactone, RU486 or vehicle and returned to the homecages. One hour later, one mouse at a time was placed in context B. After 3 minutes of free exploration, the animal was exposed to one tone for 30 seconds (presumably initiating reconsolidation) or to 6 tones repetitively with an inter-tone interval of 3 minutes (presumably initiating extinction) without exposure to any foot shock. Thirty seconds after the last tone, the mouse was returned to its homecage. Freezing behavior was scored throughout the experiment. Twenty four hours later (day 3, i.e. 48 hours after training) as well as one month later, tone-cue memory was examined again by exposing the mice to one tone in context B.

### Statistical Analysis

Freezing behavior is expressed as percentage of freezing time versus total testing time. All results are presented as mean ± SEM. Freezing behavior was scored at 24 hours (Day 2), 48 hours (Day 3) and also one month after training. Freezing behavior was therefore analyzed with repeated measures ANOVA (in SPSS 9.0), with time as a repeated measure and drug treatment as between-subject factor. Significant within-subject (time) effects and between-subject (treatment) effects were followed up using multiple mean comparions with Bonfferoni *post hoc* test. P values smaller than 0.05 were considered to be significantly different.

## References

[1] McGaugh JL (2000). Memory–a century of consolidation.. Science.

[2] de Kloet ER, Joels M, Holsboer F (2005). Stress and the brain: from adaptation to disease.. Nat Rev Neurosci.

[3] Reul JM, de Kloet ER (1985). Two receptor systems for corticosterone in rat brain: microdistribution and differential occupation.. Endocrinology.

[4] Roozendaal B (2002). Stress and memory: opposing effects of glucocorticoids on memory consolidation and memory retrieval.. Neurobiol Learn Mem.

[5] Sandi C, Rose SP (1994). Corticosterone enhances long-term retention in one-day-old chicks trained in a weak passive avoidance learning paradigm.. Brain Res.

[6] Pugh CR, Fleshner M, Rudy JW (1997). Type II glucocorticoid receptor antagonists impair contextual but not auditory-cue fear conditioning in juvenile rats.. Neurobiol Learn Mem.

[7] Oitzl MS, de Kloet ER (1992). Selective corticosteroid antagonists modulate specific aspects of spatial orientation learning.. Behav Neurosci.

[8] Zhou M, Bakker EH, Velzing EH, Berger S, Oitzl M (2010). Both mineralocorticoid and glucocorticoid receptors regulate emotional memory in mice.. Neurobiol Learn Mem.

[9] Bitran D, Shiekh M, Dowd JA, Dugan MM, Renda P (1998). Corticosterone is permissive to the anxiolytic effect that results from the blockade of hippocampal mineralocorticoid receptors.. Pharmacol Biochem Behav.

[10] Sandi C, Rose SP (1994). Corticosteroid receptor antagonists are amnestic for passive avoidance learning in day-old chicks.. Eur J Neurosci.

[11] Berger S, Wolfer DP, Selbach O, Alter H, Erdmann G (2006). Loss of the limbic mineralocorticoid receptor impairs behavioral plasticity.. Proc Natl Acad Sci U S A.

[12] de Quervain DJ, Roozendaal B, McGaugh JL (1998). Stress and glucocorticoids impair retrieval of long-term spatial memory.. Nature.

[13] de Quervain DJ, Roozendaal B, Nitsch RM, McGaugh JL, Hock C (2000). Acute cortisone administration impairs retrieval of long-term declarative memory in humans.. Nat Neurosci.

[14] Blundell J, Blaiss CA, Lagace DC, Eisch AJ, Powell CM (2011). Block of glucocorticoid synthesis during re-activation inhibits extinction of an established fear memory.. Neurobiol Learn Mem.

[15] Eisenberg M, Kobilo T, Berman DE, Dudai Y (2003). Stability of retrieved memory: inverse correlation with trace dominance.. Science.

[16] Sangha S, Scheibenstock A, Lukowiak K (2003). Reconsolidation of a long-term memory in Lymnaea requires new protein and RNA synthesis and the soma of right pedal dorsal 1.. J Neurosci.

[17] Nader K, Schafe GE, Le Doux JE (2000). Fear memories require protein synthesis in the amygdala for reconsolidation after retrieval.. Nature.

[18] Schiller D, Monfils MH, Raio CM, Johnson DC, Ledoux JE (2010). Preventing the return of fear in humans using reconsolidation update mechanisms.. Nature.

[19] Kindt M, Soeter M, Vervliet B (2009). Beyond extinction: erasing human fear responses and preventing the return of fear.. Nat Neurosci.

[20] Soeter M, Kindt M (2011). Disrupting reconsolidation: Pharmacological and behavioral manipulations.. Learn Mem.

[21] Soeter M, Kindt M (2010). Dissociating response systems: erasing fear from memory.. Neurobiol Learn Mem.

[22] Suzuki A, Josselyn SA, Frankland PW, Masushige S, Silva AJ (2004). Memory reconsolidation and extinction have distinct temporal and biochemical signatures.. J Neurosci.

[23] Nader K, Schafe GE, LeDoux JE (2000). The labile nature of consolidation theory.. Nat Rev Neurosci.

[24] Monfils MH, Cowansage KK, Klann E, LeDoux JE (2009). Extinction-reconsolidation boundaries: key to persistent attenuation of fear memories.. Science.

[25] Mamiya N, Fukushima H, Suzuki A, Matsuyama Z, Homma S (2009). Brain region-specific gene expression activation required for reconsolidation and extinction of contextual fear memory.. J Neurosci.

[26] Atkinson HC, Wood SA, Castrique ES, Kershaw YM, Wiles CC (2008). Corticosteroids mediate fast feedback of the rat hypothalamic-pituitary-adrenal axis via the mineralocorticoid receptor.. Am J Physiol Endocrinol Metab.

[27] Yang YL, Chao PK, Lu KT (2006). Systemic and intra-amygdala administration of glucocorticoid agonist and antagonist modulate extinction of conditioned fear.. Neuropsychopharmacology.

[28] Brinks V, de Kloet ER, Oitzl MS (2009). Corticosterone facilitates extinction of fear memory in BALB/c mice but strengthens cue related fear in C57BL/6 mice.. Exp Neurol.

[29] de Kloet ER, Oitzl MS, Joels M (1999). Stress and cognition: are corticosteroids good or bad guys?. Trends Neurosci.

[30] Kessels HW, Malinow R (2009). Synaptic AMPA receptor plasticity and behavior.. Neuron.

[31] Olijslagers JE, de Kloet ER, Elgersma Y, van Woerden GM, Joels M (2008). Rapid changes in hippocampal CA1 pyramidal cell function via pre- as well as postsynaptic membrane mineralocorticoid receptors.. Eur J Neurosci.

[32] Karst H, Berger S, Turiault M, Tronche F, Schutz G (2005). Mineralocorticoid receptors are indispensable for nongenomic modulation of hippocampal glutamate transmission by corticosterone.. Proc Natl Acad Sci U S A.

[33] Wiegert O, Joels M, Krugers H (2006). Timing is essential for rapid effects of corticosterone on synaptic potentiation in the mouse hippocampus.. Learn Mem.

[34] Karst H, Berger S, Erdmann G, Schutz G, Joels M (2010). Metaplasticity of amygdalar responses to the stress hormone corticosterone.. Proc Natl Acad Sci U S A.

[35] Roozendaal B, McReynolds JR, Van der Zee EA, Lee S, McGaugh JL (2009). Glucocorticoid effects on memory consolidation depend on functional interactions between the medial prefrontal cortex and basolateral amygdala.. J Neurosci.

[36] Nikzad S, Vafaei AA, Rashidy-Pour A, Haghighi S (2011). Systemic and intrahippocampal administrations of the glucocorticoid receptor antagonist RU38486 impairs fear memory reconsolidation in rats.. Stress.

[37] Herman JP, Spencer R (1998). Regulation of hippocampal glucocorticoid receptor gene transcription and protein expression in vivo.. J Neurosci.

[38] Kumar G, Couper A, O'Brien TJ, Salzberg MR, Jones NC (2007). The acceleration of amygdala kindling epileptogenesis by chronic low-dose corticosterone involves both mineralocorticoid and glucocorticoid receptors.. Psychoneuroendocrinology.

[39] Adamec R, Muir C, Grimes M, Pearcey K (2007). Involvement of noradrenergic and corticoid receptors in the consolidation of the lasting anxiogenic effects of predator stress.. Behav Brain Res.

[40] Koenig HN, Olive MF (2004). The glucocorticoid receptor antagonist mifepristone reduces ethanol intake in rats under limited access conditions.. Psychoneuroendocrinology.

[41] Zhou M, Conboy L, Sandi C, Joels M, Krugers HJ (2009). Fear conditioning enhances spontaneous AMPA receptor-mediated synaptic transmission in mouse hippocampal CA1 area.. Eur J Neurosci.

[42] Pedreira ME, Maldonado H (2003). Protein synthesis subserves reconsolidation or extinction depending on reminder duration.. Neuron.

